# Cyclin-Dependent Kinase 8 Represents a Positive Regulator of Cytomegalovirus Replication and a Novel Host Target for Antiviral Strategies

**DOI:** 10.3390/pharmaceutics16091238

**Published:** 2024-09-23

**Authors:** Debora Obergfäll, Markus Wild, Mona Sommerer, Malena Barillas Dahm, Jintawee Kicuntod, Julia Tillmanns, Melanie Kögler, Josephine Lösing, Kishore Dhotre, Regina Müller, Christina Wangen, Sabrina Wagner, Quang V. Phan, Lüder Wiebusch, Katarína Briestenská, Jela Mistríková, Lauren Kerr-Jones, Richard J. Stanton, Sebastian Voigt, Friedrich Hahn, Manfred Marschall

**Affiliations:** 1 Institute for Clinical and Molecular Virology, Friedrich-Alexander University of Erlangen-Nürnberg (FAU), Schlossgarten 4, 91054 Erlangen, Germany; debora.obergfaell@fau.de (D.O.); markus-wild@mail.de (M.W.); mona.sommerer@googlemail.com (M.S.); malena.barillas@fau.de (M.B.D.); jintawee.kicuntod@extern.uk-erlangen.de (J.K.); jul.tillmanns@fau.de (J.T.); melanie.koegler@uk-erlangen.de (M.K.); josi.loesing@fau.de (J.L.); kishore.dhotre@uk-erlangen.de (K.D.); mueller.regina@uk-erlangen.de (R.M.); christina.wangen@uk-erlangen.de (C.W.); sabrina.wagner@uk-erlangen.de (S.W.); friedrich.hahn@uk-erlangen.de (F.H.); 2 Department of Pediatric Oncology and Hematology, Charité—Universitätsmedizin Berlin, Campus Virchow-Klinikum, Augustenburger Platz 1, 13353 Berlin, Germany; qvphan@bwh.harvard.edu (Q.V.P.); lueder.wiebusch@charite.de (L.W.); 3 Richard Sherwood Laboratory, Brigham and Women’s Hospital, Harvard Medical School, 77 Avenue Louis Pasteur, Boston, MA 02115, USA; 4 Department of Microbiology and Virology, Faculty of Natural Sciences, Comenius University in Bratislava, Ilkovičova 6, 842 15 Bratislava, Slovakia; katarina.briestenska@savba.sk (K.B.); jela.mistrikova@savba.sk (J.M.); 5 Institute of Virology, Biomedical Research Center, Slovak Academy of Sciences, Dúbravská Cesta 9, 845 05 Bratislava, Slovakia; 6 Division of Infection & Immunity, School of Medicine, Cardiff University, Henry Wellcome Building, Heath Park, Cardiff CF14 4XN, UK; kerrle@cardiff.ac.uk (L.K.-J.); stantonrj@cardiff.ac.uk (R.J.S.); 7 University Clinical Center Essen (Universitätsklinikum, AöR), Institute for Virology, Virchowstr. 179, 45147 Essen, Germany; sebastian.voigt@uk-essen.de

**Keywords:** human pathogenic herpesviruses, virus-supportive cellular protein kinases, cyclin-dependent kinase 8 (CDK8), human cytomegalovirus (HCMV), regulation of viral replication, negative impact of CDK8 inhibitors, siRNA-mediated CDK8 knock-down, antiviral approach with CDK8-inhibitory small molecules, novel host-directed targeting strategies, broad-spectrum antiviral CDK8 inhibitors

## Abstract

**Background**. Cyclin-dependent kinase 8 (CDK8) is a multifaceted regulator and represents a catalytic component of the transcriptional Mediator complex. CDK8 activity, on the one hand, increases transcriptional elongation by the recruitment of Mediator/super elongation complexes, but, on the other hand, negatively regulates CDK7-controlled transcriptional initiation through inactivating cyclin H phosphorylation. Recently, these combined properties of CDK8 have also suggested its rate-limiting importance for herpesviral replication. **Objectives**. In this paper, we focused on human cytomegalovirus (HCMV) and addressed the question of whether the pharmacological inhibition or knock-down of CDK8 may affect viral replication efficiency in cell culture models. **Methods**. A number of human and animal herpesviruses, as well as non-herpesviruses, were used to analyze the importance of CDK8 for viral replication in cell culture models, and to assess the antiviral efficacy of CDK8 inhibitors. **Results**. Using clinically relevant CDK8 inhibitors (CCT-251921, MSC-2530818, and BI-1347), HCMV replication was found strongly reduced even at nanomolar drug concentrations. The EC_50_ values were consistent for three different HCMV strains (i.e., AD169, TB40, and Merlin) analyzed in two human cell types (i.e., primary fibroblasts and astrocytoma cells), and the drugs comprised a low level of cytotoxicity. The findings highlighted the following: (i) the pronounced in vitro SI values of anti-HCMV activity obtained with CDK8 inhibitors; (ii) a confirmation of the anti-HCMV efficacy by CDK8–siRNA knock-down; (iii) a CDK8-dependent reduction in viral immediate early, early, and late protein levels; (iv) a main importance of CDK8 for viral late-stage replication; (v) several mechanistic aspects, which point to a strong impact on viral progeny production and release, but a lack of CDK8 relevance for viral entry or nuclear egress; (vi) a significant anti-HCMV drug synergy for combinations of inhibitors against host CDK8 and the viral kinase vCDK/pUL97 (maribavir); (vii) finally, a broad-spectrum antiviral activity, as seen for the comparison of selected α-, β-, γ-, and non-herpesviruses. **Conclusions**. In summary, these novel data provide evidence for the importance of CDK8 as a positive regulator of herpesviral replication efficiency, and moreover, suggest its exploitability as an antiviral target for novel strategies of host-directed drug development.

## 1. Introduction

Human cytomegalovirus (HCMV) is a worldwide-distributed human β-herpesviral pathogen with regional seropositivity between 40% and 95%. HCMV is able to establish life-long latent infection, which typically remains asymptomatic in the immunocompetent host. In immunocompromised individuals, however, HCMV infection often develops severe or even life-threatening symptoms [1]. These high-risk groups include post-transplant recipients, AIDS patients, or individuals under cancer or immunosuppressive therapy. Even more importantly, congenital HCMV infection (cCMV) of the immunonaïve host during pregnancy may lead to the appearance of severe cytomegalovirus inclusion disease (CID) and developmental defects in the neonate, such as sensorineural hearing loss, intellectual disability, or microcephaly [2,3]. The symptomatic range of cCMV has been assigned to approx. 20–25% of all infected newborns. The currently available drugs for the treatment of HCMV infections include pharmacological inhibitors of the viral DNA polymerase (i.e., ganciclovir, its oral prodrug valganciclovir, cidofovir, and foscarnet), the viral terminase (letermovir), and the viral protein kinase (maribavir). Despite the great benefit these approved anti-HCMV drugs entail for the prevention of HCMV disease, potentially all of these show limitations in their applicability, i.e., the development of viral drug resistance, in part the occurrence of side effects particularly under long-term treatment including restrictions of oral bioavailability, as well as a lack of approval for clinical use during pregnancy [4,5]. Thus, additional options of anti-HCMV therapy, including, to date, unexploited targeting strategies and new classes of inhibitors, are urgently needed. In this regard, a current targeting option is based on the potential use of host-directed antivirals (HDAs), in addition to the abovementioned group of established direct-acting antivirals (DAAs) [5,6,7,8,9]. Moreover, recent studies from our group and other investigators demonstrated a remarkable level of statistical drug synergies in anti-HCMV combination treatments with DAAs and HDAs [10,11,12,13,14]. As far as the latter topic is concerned, pharmacological inhibitors of cyclin-dependent kinases (CDKs) could be characterized as very potent anti-HCMV investigational candidates and suggested CDK-directed targeting as a next-generation developmental strategy towards new antiviral small molecules [12,14,15,16]. In this ongoing line of research, both host CDKs, such as CDK7 (i.e., using the selective developmental inhibitors LDC4297 [17] and QRS6/7/9 [18]), as well as the viral ortholog vCDK/pUL97 (i.e., using the clinically approved maribavir/MBV [5,19,20]) were nominated as highly promising antiviral targets. These studies identified a statistically significant synergistic antiviral effect through in vitro or in vivo combination treatments, using inhibitors directed to host CDKs and vCDK/pUL97 [12,13,14]. Also, novel examples of HCMV-specific drug synergy have been demonstrated by exclusive HDA treatment combinations, i.e., using inhibitors against CDK2, CDK7, CDK8, and CDK9, or triple inhibitor combinations between CDKs and vCDK/pUL97 [12,14]. In particular, CDK8 is a complex regulator with manifold activities. On the one hand, after RNAPII has departed from the promoter, the associated CDK8 recruits the core Mediator and super elongation complex (SEC), thus leading to increased transcriptional elongation. On the other hand, CDK8 is able to phosphorylate cyclin H in a manner that leads to a deactivation of CDK7–cyclin H–MAT1, thus preventing the initiation of transcription [21]. Interestingly, these activities have recently been considered as rate-limiting steps of herpesviral replication efficiency, in particular of HCMV replication [22].

In the present study, we focused on the role of CDK8 as a putatively novel anti-HCMV drug target, since a few points of our preliminary investigations appeared striking. First, CDK8 belongs to the transcriptional group CDKs (together with CDK7, CDK9, and potentially some others; [23]). Within this group, specific functions may be compensated between the related CDKs, under conditions of functional impairment or CDK-specific pharmacological inhibition. This compensation, however, may primarily concern their cellular functions, but probably not their specialized virus-supportive functions. Second, several CDK8 inhibitors have already been developed and thus reached a level of clinical investigations in the area of cancer therapy [24]. This encouraged their further characterization in the field of antiviral applicability. Third, our previous experimental work, which focused on the comparative use of various CDK inhibitors in antiviral assessments, strongly suggested that CDK8 represents a broadly acting coregulatory factor in herpesviral replication.

The novel findings of the present study strongly underline this concept. An important step of validation was provided by the use of CDK8-dowmodulatory siRNAs, which demonstrated a negative impact on viral replication. Importantly, a series of selective and highly potent CDK8 inhibitors showed a marked pattern of antiviral activity, which strengthened our earlier statement about the central importance of CDKs as novel host targets for antiviral drug development [12]. The data of the present work illustrate this important aspect of CDK8, i.e., the positive regulation of viral replication as demonstrated for HCMV and other human and animal pathogenic viruses, by using various experimental approaches. The mechanistic basis is still a subject of detailed analyses, and the current state of the data points to a multifunctional role of CDK8-mediated proviral regulation. In essence, the study argues for considering CDK8, related CDKs, and their regulated pathways, for future antiviral targeting strategies and pharmacological compound developments.

## 2. Materials and Methods

### 2.1. Cells and Viruses

Primary human foreskin fibroblasts (HFFs; obtained from clinical samples at Children’s Hospital in Erlangen, Germany), guinea pig embryonic fibroblasts (GPEFs; generously provided by Mark R. Schleiss from the University of Minnesota in Minneapolis, MN, USA), and rat embryonic fibroblasts [25] were cultivated in Eagle’s Minimal Essential Medium (MEM) supplemented with 1 × GlutaMAX™ (both from Thermo Fisher Scientific, Waltham, MA, USA), 10 µg/mL gentamicin, and 10% fetal bovine serum (FBS; Capricorn, Ebsdorfergrund, Germany). Human glioblastoma–astrocytoma cell line (U373) and mouse embryonic fibroblasts (MEFs), both obtained from ATCC (Manassas, VA, USA), and Vero cell sublineage 76 [26], were cultivated in Dulbecco’s modified Eagle’s medium (DMEM) supplemented with 1 × GlutaMAX™, 10 µg/mL gentamicin, and 10% fetal bovine serum. All cultured cells were maintained under the conditions of 37 °C temperature, 5% CO_2_, and 80% humidity. The regular monitoring for the absence of mycoplasma contamination was conducted using Lonza™ Mycoalert™ (Thermo Fisher Scientific, Waltham, MA, USA). Cultured cell-based viral replication assays were performed by the use of the HCMV strain AD169 (variant U.K.), the recombinant HCMVs AD169-GFP expressing green fluorescent protein [27], TB40 IE2-YFP and TB40-FLuc expressing an IE2-YFP fusion protein or firefly luciferase (FLuc) [28,29,30], and Merlin-GFP [31]. Furthermore, the non-human CMVs MCMV-C3X-GFP (recombinant MCMV expressing GFP; [32], GPCMV-GFP (recombinant guinea pig CMV expressing GFP; [33]), RhCMV-EGFP (recombinant rhesus macaque CMV expressing GFP; [34]), rat CMV (RCMV; [25]), RCMV-mCherry [35], and chimpanzee CMV (ChCMV; [36]) were used. Additionally applied viruses, for the purpose of comparative antiviral settings, were as follows: human herpesvirus type 6A (HHV-6A-GFP, in J-Jhan T cells, kindly provided by Benedikt Kaufer, FU Berlin, Germany; [37]); herpes simplex virus type 1 (HSV-1 166v VP22-GFP, in HFFs, kindly provided by Peter O’Hare, London, UK; [18]); varicella zoster virus (VZV Oka-GFP, in HFFs, kindly provided by Benedikt Kaufer, FU Berlin, Germany; [18,38]); Kaposi’s sarcoma-associated herpesvirus (KSHV rKSHV.219-GFP/RFP, in iSLK.219 carrier cells; [18]); Epstein–Barr virus (EBV Akata-GFP produced in Akata-BX1-GFP carrier cells, infections performed in Raji-derived R81GFP reporter cells; [18,39]); murine herpesvirus 68 (and MHV-68-Luc, in Vero cells; [37,40]); human adenovirus (HAdV-C5 DBP mneongreen, in A549 cells; [41]), and human polyomavirus (JCPyV strain Mad-4, in COS-7 cells; [42]).

### 2.2. Antiviral Compounds

Antiviral compounds were obtained from MedChemExpress, Monmouth Junction, NJ, USA (maribavir, BI-1347, CCT-251921, MSC-2530818, and SEL120). Sterile DMSO (Sigma Aldrich, St. Louis, MO, USA) was used to prepare stock solutions, with aliquots stored at −20 °C.

### 2.3. Antiviral Drug Assessment of Recombinant HCMV Infection in Primary Cells

HFFs were seeded at 1.35 × 10^4^ cells/well in 96-well cell culture plates and infected on the following day with HCMV AD169-GFP, TB40 IE2-YFP, TB40-FLuc, or Merlin-GFP [27,30,31,43], a dilution that resulted in approx. 25% positive cells at 7 d p.i. (i.e., 1 × TCID_25_/7d). Immediately after virus adsorption, the cells were treated with antiviral compounds or solvent control (DMSO). Infections were performed in biological quadruplicates, or as indicated for individual experiments. The cells were fixed by the addition of 150 µL 10% formalin/well 7 d p.i. (Merlin-GFP 14 d p.i.), and washed with PBS. For GFP or YFP quantitation, the Victor X4 microplate reader (PerkinElmer, Waltham, MA, USA) was primarily used, or as a very sensitive alternative, the ImageXpress^®^ Pico device (Molecular Devices LLC, San Jose, CA, USA). Mean EC_50_ values of antiviral efficacy were calculated on the basis of three experimental replicates or as indicated (measurement of fluorescence values in quadruplicate), and percentage values were provided relative to the solvent control (DMSO).

### 2.4. Antiviral Drug Assessment of Animal CMV Infections in Primary Cells

Similarly, 2 × 10^4^ primary embryonic guinea pig fibroblasts GPEFs/96-well, or 1.35 × 10^4^ HFFs/96-well, or 2 × 10^4^ rat REFs/96-well, or 2 × 10^4^ murine MEFs/96-well, were cultivated for infection with GPCMV-GFP, RhCMV-GFP, ChCMV, RCMV-mCherry, or MCMV-GFP, respectively (all approx. MOI of 0.01 referring to 1 × TCID_25_), and were used for the assessment of antiviral drug activity. To this end, cells were fixed 7 d p.i. (RCMV-mCherry 4 d p.i.), before GFP or mCherry expression was quantified using the Victor X4 microplate reader, or other viral replication markers were measured as explained for the individual experiments. ChCMV replication in HFFs was monitored and quantified by the use of Anti-Cytomegalovirus Antibody Alexa Fluor™ 488. For immunofluorescence staining with Anti-Cytomegalovirus Antibody Alexa Fluor™ 488 (Sigma Aldrich, St. Louis, MO, USA; solution in PBS with 1% FCS), the infected cells were fixed at 7 d p.i. with 10% formalin and permeabilized with 0.2% Triton-x-100. Cell nuclei were counterstained using Höchst dye to be measured using the ImageXpress Pico (Molecular Devices, LLC, San Jose, CA, USA). Mean EC_50_ values of antiviral efficacy were calculated on the basis of three experimental replicates or as indicated (measurement of fluorescence values in quadruplicate), and percentage values were provided relative to the solvent control (DMSO).

### 2.5. Antiviral Drug Assessment of α-, β-, and γ-Herpesviral and Non-Herpesviral Infections

A variety of antiherpesviral and non-herpesviral drug activities were assessed by the cell culture-based viral replication systems, as described elsewhere. For viruses, recombinants, permissive cells, methods, and references (HSV-1, VZV, HHV-6A, KSHV, EBV, MHV-68, HAdV, and JCPyV), see Section 2.1. In specific terms for KSHV, doxycycline (dox)-inducible iSLK.219 cells, harboring the double reporter-expressing rKSHV.219 [44,45], were used in antiviral drug assessments as described previously [18]: the latency phase of rKSHV.219 is expressed through the GFP signal, while the dox-reactivated productive phase (inducible viral transactivator Rta) is expressed through the RFP signal, so that iSLK.219 cells, cultivated in the 96-well format, were induced to lytic replication by the addition of 0.25 µg/mL dox in the presence of antiviral compounds treatment. After incubation at 37 °C for 3 days, iSLK.219 cells were fixed with 10% formalin, washed with PBS, and used for a quantitation of RFP reporter signal by PicoMD counting (ImageXpress^®^ Pico device, Molecular Devices LLC, San Jose, CA, USA). For adenovirus, A549 cells were seeded at 5 × 10^4^ cells/96-well, used for infection at the following day with recombinant HAdV-C5 DBP, and treated with compounds or solvent control. Cells were fixed with 10% formalin, before the expressed mneongreen reporter was measured using the Victor X4 microplate reader. For polyomavirus, COS-7 cells were seeded at 5 × 10^4^ cells/96-well, used for infection at the following day with JCPyV Mad-4 and treated with compounds or solvent control. Infectious supernatants were collected at the time points indicated, to be used in a quantitative, virus-specific PCR (JCPyV qPCR). All abovementioned antiviral EC_50_ values refer to measurements in triplicate.

### 2.6. Neutral Red (NR) Uptake and Alamar Blue (AB) Cell Viability Assays

To address cell viability after compound treatment, a NR uptake assay was performed. Uninfected cells were seeded at equal numbers as for antiviral replication assays and treated with compounds or solvent control. The treatment was performed for HFFs (7 d), U373 (14 d), A549 (3 d), J-Jhan (14 d), and non-dox-induced iSLK.219 cells (3 d). At the end of the treatment period, 40 µg/mL of Neutral Red (NR) was added and incubated for 2–4 h at 37 °C, before the NR-containing supernatant was removed. Cells were destained using a solution containing 50% ethanol, 1% acetic acid, and 49% water. NR fluorescence was quantified using the Victor X4 microplate reader (PerkinElmer, Waltham, MA, USA) (560/630 nm). For the AB cell viability assay, which was also used to quantify virus-induced cytopathogenicity (particularly in the case of GPCMV in GPEFs), cells were seeded at equal numbers as for antiviral replication assays and incubated with a medium containing 25 ng/mL AB (resazurin; Ann Arbor, MI, USA), incubated for 6 h at 37 °C, and used for measurement identical with NR.

### 2.7. Anti-HCMV Drug Interaction Assessment via Loewe Additivity Fixed-Dose Assay

Loewe additivity fixed-dose assay was performed using an adapted protocol of the HCMV-GFP-based replication system, as described previously [17,27]. In these settings, cells were treated either with a single compound, compound combinations, or solvent control. Antiviral efficacy (mean of quadruplicate measurements of biological duplicates) was expressed as the percentage of solvent control, as entered into the CompuSyn software (Version 1.0 [46]; ComboSyn, Inc., Paramus, NJ, USA). Only experiments with an r value > 0.90 and EC_50_ values close to previously determined concentrations were accepted.

### 2.8. Western Blot (Wb) Analysis of HCMV Protein Production

Western blot (Wb) analyses were performed as previously described [17] using total lysates prepared from infected and mock-infected HFFs. For this purpose, HFFs were seeded at 3 × 10^5^ cells/6-well and used for infection with HCMV AD169, or other viruses as indicated, then treated with compounds or solvent control. At the given time points of infection, the total lysates were prepared and used for separation on standard SDS-PAGE/Wb procedures. For Wb staining, antibodies specific for CDK8 (A302-501A, Bethyl Laboratories, Montgomery, TX, USA); viral proteins such as mAb-IE1, mAb-UL44, mAb-pp28, and mAb-MCP (kindly provided by S. Jonjic and T. Lenac Rovis, Rijeka, Croatia); as well as mAb-β-actin as a house-keeping protein and loading control (A5441, Sigma Aldrich, St. Louis, MO, USA) were used, followed by incubation and staining with the appropriate HRP-conjugated secondary antibodies.

### 2.9. Small Interfering RNA (siRNA) Transfection and Assesment of Viral Replication Efficiency

For siRNA transfection, 3 × 10^5^ HFFs/6 wells were cultivated and applied for siRNA transfection, performed by the use of 9 µL Lipofectamine™ RNAiMAX (Thermo Fisher Scientific, Waltham, MA, USA) and 10 pmol siRNA in Opti-MEM™, or by Lipofectamine™ 3000 (Invitrogen, Waltham, MA, USA). At 2 d post-transfection, cells were infected with HCMV AD169 (MOI 0.1), before cells were harvested at 7 d p.i. for the assessment of viral protein expression via Wb analysis.

### 2.10. Indirect Immunofluorescence (IF) Analysis and Confocal Laser-Scanning Microscopy

In order to investigate viral protein expression and localization, 3 × 10^5^ HFFs were seeded in 6-well plates one day prior to infection with HCMV AD169-UL50-HA (MOI 0.05). After 90 min of viral absorption, cells were treated with 0.5 µM MBV, 5 nM CCT, 2 nM MSC or 0.7 nM BI-1347. The infected cells were fixed at 7 d p.i., before indirect immunofluorescence staining was performed for viral IE1, pUL50, and pUL53, and signals were evaluated by confocal laser-scanning microscopy (Leica Microsystems, TCS SP5, Wetzlar, Germany). The counterstaining of the nuclei was performed with DAPI.

### 2.11. HCMV-Specific and JCPyV-Specific Quantitative Polymerase Chain Reactions (qPCRs)

For HCMV, viral genomic copy numbers were determined from the supernatant of infected HFFs by a HCMV IE1-specific quantitative real-time PCR (qPCR), as described previously [47]. For viral replication kinetics, cells were infected at MOI of 0.01, and supernatant samples were collected at the indicated time points. The DNase treatment of the virus-containing aliquots was performed according to the manufacturer’s protocol (04716728001, Sigma Aldrich). The PCR standard contained 10^2^ HCMV DNA copies and reached the cycle threshold at approx. cycle number 38 (defined as the limit of detection). For JCPyV, viral genomic copy numbers were determined from the supernatant of infected COS-7 cells by a JCPyV Ltag-specific qPCR (genomic region nt 4289–4375), as described elsewhere [42]. The primers 5′ JCV-F, 5′-AGA GTG TTG GGA TCC TGT GTT TT-3′, and 3′ JCV-R, 5′-GAG AAG TGG GAT GAA GAC CTG TTT-3′; 5′FAM/3′TAMRA probe, 5′-TCA TCA CTG GCA AAC ATT TCT TCA TGG C-3′ were used. Shortly before qPCR, the supernatants were diluted in distilled H_2_O (1:100 dilution) and heat-denatured at 95 °C for 5 min.

## 3. Results and Discussion

### 3.1. Assessment of Anti-HCMV Activity of CDK8 Inhibitors 

Recently, we demonstrated the supportive function of CDK8 activity for HCMV replication and the antiviral activity of the selective CDK8 inhibitor SEL120 [12]. In comparison to related small molecules targeting either host- or virus-encoded CDKs (CDK2, CDK7, CDK8, or vCDK/pUL97), the CDK8 inhibitor SEL120 showed a promising profile of HCMV-inhibitory properties, with an EC_50_ value of 0.079 ± 0.001 µM, thus ranging at highly promising nanomolar concentrations (Table 1). Seen apart from the monoselective CDK7 inhibitor, which has been profoundly characterized by previous studies [12,13,14,17,28,48], especially the CDK8 inhibitor demonstrated a highly interesting profile of anti-HCMV efficacy in the fibroblast-based infection model, superior to the latest approved clinical drug maribavir (MBV).

**Figure 1:**
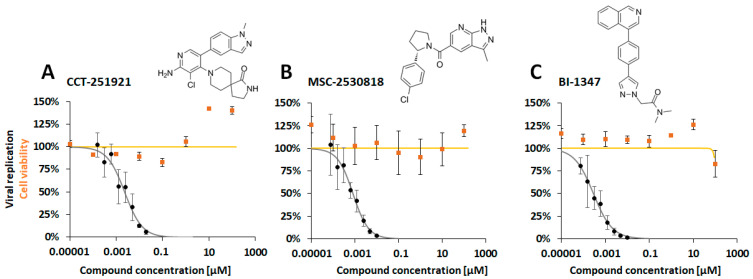
Anti-HCMV activity was demonstrated for three CDK8-inhibitory developmental small molecules: (**A**) CCT-251921, (**B**) MSC-2530818, and (**C**) BI-1347. The assessment utilized a GFP replication assay employing HCMV AD169-GFP for infecting HFFs. The compounds were administered immediately p.i., starting at a concentration of 50 nM, followed by eight consecutive five-fold dilution steps. Cells were fixed at 7 d p.i. for quantitative GFP fluorometry. Additionally, cell viability in uninfected cells was determined through NRA (curves in orange). The presented values represent the mean ± SD of triplicate (NRA) or quadruplicate (GFP), with the data representing one experiment out of at least three independent replicates.

On this basis, we then attempted to develop this understanding by including three novel CDK8 inhibitors that are currently investigated at the clinical developmental stage [49,50] (see chemical formula in Figure 1). Using the established antiviral GFP-based HCMV replication assay, we demonstrated a nanomolar inhibitory activity of CCT-251921 (CCT), MSC-2530818 (MSC), and BI-1347 (BI) in HCMV-infected primary human fibroblasts (HFFs). Very pronounced, concentration-dependent antiviral profiles were obtained for all three CDK8 inhibitors (Figure 1A–C) against HCMV AD169-GFP replication in the absence of cytotoxicity. The EC_50_ values ranged at nanomolar concentrations, with 3.90 ± 2.00 nM, 1.21 ± 0.77 nM, and 0.93 ± 0.67 nM for CCT, MSC, and BI, respectively, in HCMV AD169-GFP-infected HFFs (Table 2, upper left). This clearly supported our notion that CDK8 inhibition might serve as an efficient anti-HCMV-targeting strategy. As a next step, we compared three strains of HCMV in the infection of two different cell types. In all cases, the CDK8 inhibitors CCT, MSC, and BI showed pronounced antiviral activity, with EC_50_ values in a range between 19.33 ± 21.00 nM and 0.54 ± 0.99 nM (Table 2, all panels). This antiviral efficacy was similarly observed for the fully HCMV-permissive HFFs (Table 2, upper part), and the semi-permissive U373 cells (Table 2, lower part). Thus, the findings confirm the strong anti-HCMV potency of CDK8 inhibitors as applied under various conditions of HCMV infection.

**Figure 2:**
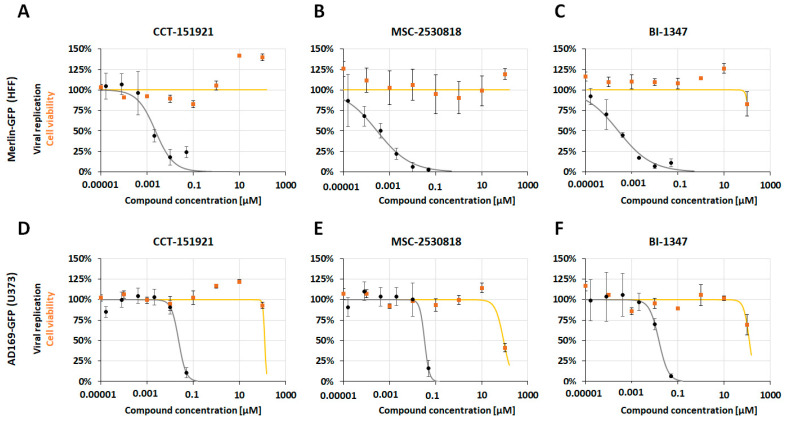
Anti-HCMV activity was demonstrated for three CDK8-inhibitory developmental small molecules: For Merlin-GFP in HFF cells (**A**) CCT-251921, (**B**) MSC-2530818, and (**C**) BI-1347, and AD169-GFP in U373 cells (**D**) CCT-251921, (**E**) MSC-2530818, and (**F**) BI-1347. The experiment utilizes a GFP replication assay with HCMV AD169-GFP or Merlin-GFP to infect HFF or U373 cells. The compounds were administered immediately post-infection (p.i.), beginning at a concentration of 50 nM, followed by eight five-fold dilution steps. Cells were fixed at 7 days post-infection (d p.i.) for quantitative GFP fluorometry. Additionally, cell viability in uninfected cells was assessed using the NRA (curves in orange). The values presented are the mean ± SD of triplicate (NRA) or quadruplicate (GFP) measurements, with data representing one experiment out of at least three independent replicates.

In a next step, we addressed the question of whether this pronounced level of antiviral activity of the selective CDK8 inhibitors did not exclusively hold true for the standard strain AD169 on fibroblasts, but also for neuronal-type U373 cells (of glioblastoma–astrocytoma origin) and for more clinically relevant strains of HCMV, like Merlin and TB40. To this end, the equivalent settings described for Figure 1 were now applied using HCMV AD169-GFP, TB40-YFP, and Merlin-GFP, each on both HFFs and U373 cells (Figure 2, Table 2, Figure S1). As an important finding, the antiviral activities of the CDK8 inhibitors CCT, MSC, and BI were measured for all these experimental settings, all ranging in the nanomolar spectrum of drug concentrations. Although the individual EC_50_ values showed some minor differences between the compared viruses and cells (Figure 2, Table 2, Figure S1), the general inhibitory potential of CDK8 inhibitors against HCMVs was confirmed by these experiments.

### 3.2. Analysis of the Effect of CDK8 Inhbititor Treatment on HCMV Protein Expression

**Figure 3:**
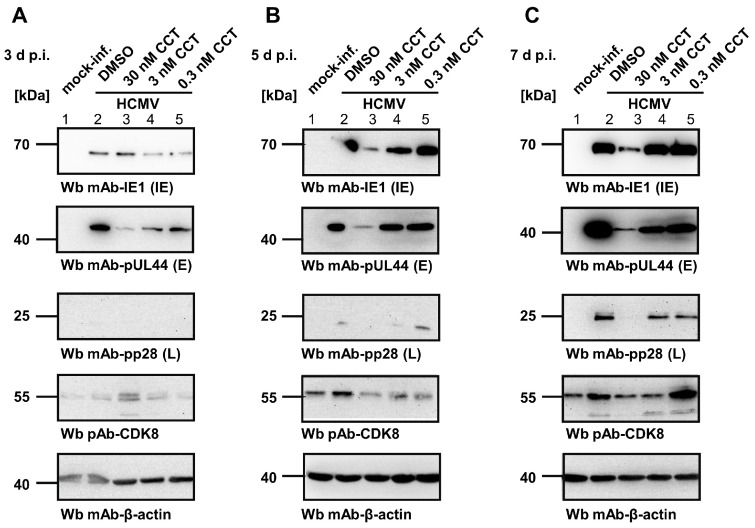
Analysis of the antiviral effect of the CCT CDK8 inhibitor. HFFs were cultivated in 6-well plates and infected with HCMV AD169 at MOI of 0.1. Infected cells were treated with 30 nM, 3 nM or 0.3 nM CCT. Mock-infected cells and DMSO-treated infected cells were used as control samples. Cells were harvested at 3 d (**A**), 5 d (**B**), and 7 d p.i. (**C**) for the preparation of total lysates, to be analyzed by SDS-PAGE/Wb procedures. Wb antibody staining was performed for viral IE1p72, pUL44, and pp28, thus representing viral immediate early (IE), early (E), and late (L) marker proteins, respectively. In addition, cellular CDK8 (drug target) as well as β-actin (house-keeping protein and loading control) were stained in parallel.

To address the question of at which stage of the HCMV replication cycle the CDK8 inhibitors exert their main antiviral activity, we performed a Wb analysis of viral protein expression. For this purpose, HFFs were infected with HCMV AD169 at MOI of 0.1, treated with the CCT compound at three different concentrations (30 nM, 3 nM, and 0.3 nM), before samples were collected at 3 d, 5 d, and 7 d p.i., thus representing the first, second, and third round of viral replication, respectively (Figure 3, panels A–C). Wb staining was performed against the typical markers of viral immediate early (IE), early (E), and late (L) stages of gene expression, as indicated. The expression of CDK8 under the CCT treatment was monitored in parallel, thereby showing an upregulation by HCMV infection (Figure 3A–C), lanes 2; DMSO) compared to the uninfected controls (Figure 3A–C, lanes 1; mock-inf.). The Wb stainings also showed some minor variation of CDK8 quantities between the samples, but without a clear drug-specific impact (note, that CDK8 expression was found lowest under the highest CCT concentration in panels B–C, lanes 3, which may be explained by the general impact of CDK8 on transcription efficiency). Importantly, the viral proteins showed substantially reduced levels compared to the infection DMSO control (Figure 3A–C, column 2), in particular responding to the increase in CCT concentrations (columns 5, 4, and 3). The inhibitory activity of the drug was already detectable at the IE level of proteins, which then translated into a more drastic effect at the E level, and a complete inhibition at the L level for 30 nM of CCT (see column 3 in panels B–C; upper Wbs for viral IE1p72, second upper Wbs for pUL44, and middle Wbs for pp28). This finding strongly suggests an antiviral mode of action (MoA) of the CDK8 inhibitors that increases in efficacy over the time of infection, with a most drastic inhibitory effect expressed at the late stage of viral replication.

### 3.3. Analysis of Viral Protein Production after siRNA-Mediated CDK8 Knock-Down in HCMV-Infected Cells

**Figure 4:**
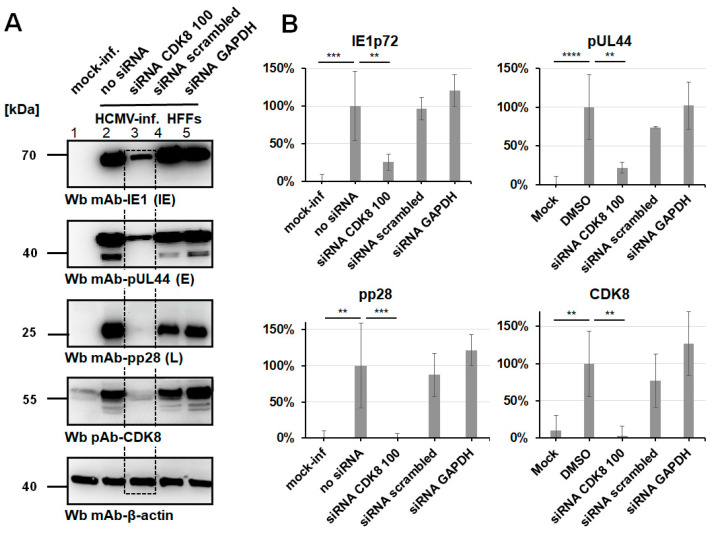
Analysis of transfection-mediated intracellular CDK8-specific siRNA knock-down (KD) in primary fibroblasts. (**A**) The CDK8-specific siRNA KD was specifically reduced, under slightly optimized conditions (Lipofectamine™ RNAiMAX), using the siRNAs CDK8 100 at a final concentration of 10 pmol/well. HFFs were cultivated in 6-well plates and infected with HCMV AD169 at MOI of 0.1 at two d post-siRNA transfection (see KD effect highlighted by dashed box). As control samples, mock-infected cells, mock-transfected (no siRNA) cells, a non-specific siRNA (scrambled), and a GAPDH-specific siRNA were used. Cells were harvested at 7 d p.i. for the preparation of total lysates, to be analyzed by SDS-PAGE/Wb procedures. (**B**) Densitometric analysis was performed in quadruplicate measurements (SDS-PAGE/Wbs in duplicate, densitometry in duplicate) using AIDA image analyzer, and statistical evaluation was conducted using ANOVA followed by Bonferroni’s correction method for multiple comparison (** *p* ≤ 0.01, *** *p* ≤ 0.001, **** *p* ≤ 0.0001).

In order to verify the relevance of CDK8 for HCMV replication, we applied a knock-down strategy. The transfection of synthetic, target-specific siRNAs into HCMV-infected cells was performed to confirm the CDK8-directed antiviral effect seen with CDK8 inhibitory small molecules. To this end, three siRNAs with specificity for CDK8 were used for transfection of HCMV-infected HFFs (siRNAs CDK8 100, CDK8 101, and CDK8 102; Lipofectamine™ 3000 transfection; Figure S5). At 7 d p.i., virus-specific Wb analysis was performed to visualize the effect of CDK8 knock-down (KD) on the production of viral proteins of the replicative stages IE, E, and L (Figure 4). A pronounced inhibitory effect was observed for siRNA CDK8 100, which was moderate at the IE and E stages (IE1p72, pUL44) and was very strong at the viral L stage (MCP, pp28; Figure 4A). In the control panel of CDK8 staining, siRNA CDK8 100 showed an almost complete CDK8 KD (Figure 4A, lane 4 as marked by framing), which was consistent with the result of inhibited viral protein synthesis. This finding supports the concept of CDK8 playing a virus-supportive role in HCMV replication, as demonstrated by a second, independent experimental approach, using the most potent siRNA. To this end, the siRNA CDK8 100 that showed specificity for CDK8 was used under slightly optimized conditions (Lipofectamine™ RNAiMAX) for the transfection of HCMV-infected HFFs. At 7 d p.i., a virus-specific Wb analysis was performed to visualize the effect of CDK8 KD, again on the production of viral proteins of the replicative stages IE, E, and L (Figure 4A). An even more pronounced inhibitory effect was observed for siRNA CDK8 100, as clearly expressed at the IE and E stages (IE1p72, pUL44), and again most strongly at the viral L stage (pp28; Figure 4A, column 3, middle panel). In the control of CDK8 staining, siRNA CDK8 100 showed an almost complete CDK8 KD (Figure 4A, column 3, second to last panel), thus strongly confirming our previous result. For this setting, a densitometric quantitation of Wb signals was performed (mean values of quadruplicate determination ± SD; Figure 4B). Statistical significance was obtained for the siRNA-mediated reduction in protein signals in all cases analyzed, i.e., IE1p72, pUL44, pp28, and likewise on CDK8 as the target of KD. In comparison, the marginal effects of control transfections performed with siRNA scrambled and siRNA GAPDH did not show statistical significance (Figure 4B). In addition, an identical experimental approach was performed on HCMV-infected U373 cells, using siRNA-mediated KD as described (Lipofectamine™ RNAiMAX). Similar to HFFs, a siRNA CDK8-mediated antiviral effect could be detected for U373 cells, with greatest inhibitory impact on viral L stage of replication (Figure S6). The efficacy of CDK8 KD was demonstrated by the use of additional time-point samples of 2, 5, 7, and 9 d post-transfection (Figure S6C). Thus, all experiments performed with the siRNA approach support our statement of the important role that CDK8 exerts on HCMV replication.

### 3.4. Mechanistic Aspects of the CDK8-Specific Antiviral Effect

**Figure 5:**
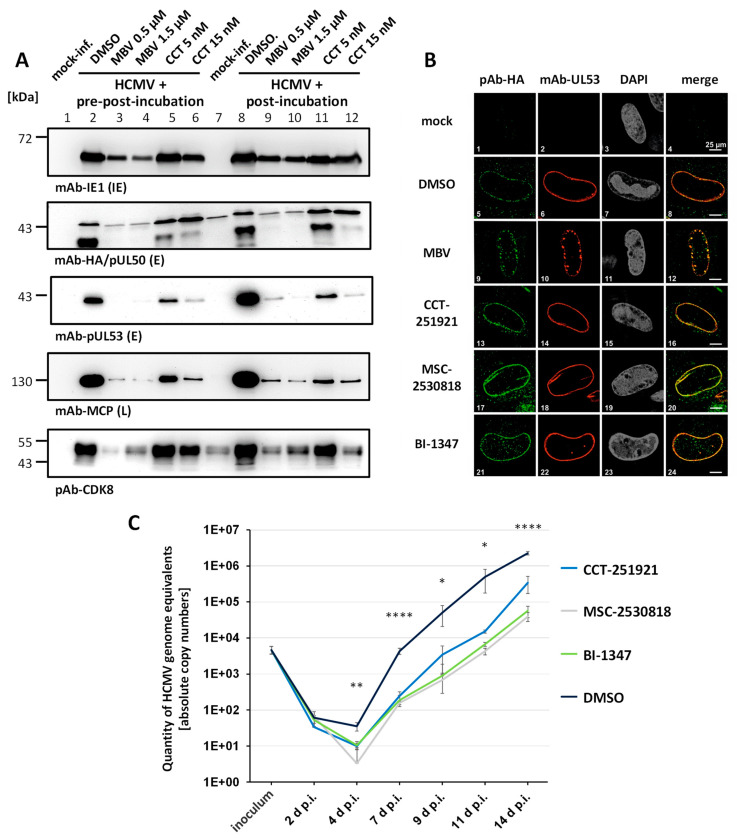
Mechanistic characteristics of the antiviral activity of CDK8 inhibitors. (**A**) An analysis of virus entry in the presence of CDK8 inhibitors was performed by comparing pre-post-incubation and post-incubation conditions of drug treatment. HFFs were infected with recombinant HCMV AD169 expressing an HA-tagged pUL50 (AD169-UL50-HA) at MOI of 0.01. For vCDK and CDK8 inhibitor treatments, two different setups were used. One set of cells was treated with MBV (0.5 µM and 1.5 µM) or CCT (5 nM and 15 nM) before, during, and after infection (pre-post). The other set was exclusively treated 90 min after virus absorption (post). Western blot staining was performed for IE1p72, pUL50-HA, pUL53, MCP, and CDK8. (**B**) The typical nuclear rim localization of viral NEC proteins was analyzed under CDK8 inhibitor treatment. HFFs were cultivated in 6-well plates on cover slips, and used for infection with HCMV AD169-UL50-HA at MOI of 0.05. After 90 min of virus absorption, cells were treated with 0.5 µM MBV, 5 nM CCT, 2 nM MSC, or 0.7 nM BI. Infected cells were fixed at 7 d p.i., and IF staining was performed for viral pUL50-HA and pUL53 to be analyzed by confocal imaging. Counterstaining of the nuclei (DAPI) is indicated, and a merge of pUL50-HA and pUL53 signals is provided on the right. (**C**) The reduction in virus progeny production and release was quantitated under CDK8 inhibitor treatment. For this purpose, HFFs were infected with AD169-UL50-HA at MOI of 0.01 and treated with 5 nM CCT, 2 nM MSC, 0.7 nM BI, or DMSO in the control. Supernatant samples were taken at 2, 4, 7, 9, 11, and 14 d p.i., to perform virus-specific qPCR analysis. Statistical evaluation was conducted using an ANOVA followed by Bonferroni’s correction method for multiple comparison (* *p* ≤ 0.05, ** *p* ≤ 0.01, **** *p* ≤ 0.0001).

For addressing the question of whether CDK8 inhibitors may exert specific mechanistic alterations in the HCMV replication cycle, three different parameters were analyzed (Figure 5). First, we measured putative inhibitory effects on virus entry and the efficiency of viral intracellular uptake. For this purpose, HCMV-infected HFFs were subjected to different conditions of drug treatment. The CDK8 inhibitor CCT-151921 (CCT) was either used for a pre-incubation of cells, prior to virus infection (pre-post-incubation), or to a normal condition of post-infection treatment (post-incubation), in which a partially effective concentration of the drug was applied immediately after the virus adsorption phase. In both cases, drug treatment was continued for the entire period of cultivation of HCMV-infected cells (7 d p.i.). The inhibitor of viral protein kinase pUL97, maribavir (MBV, a late-stage inhibitor of HCMV replication), was used as a reference drug (Figure 5A). A comparative evaluation of the Wb staining illustrated, on the one hand, an antiviral drug effect for both CCT and MBV, which was moderate at the IE level, intermediate at the E level, and strong at the viral L stage of replication (Figure 5A, upper four panels). Both antiviral compounds also effected a partial block of the virus-induced upregulation of CDK8, which was more pronounced for MBV compared to CCT (Figure 5A, lowest panel, columns 3–6 and 9–12 to be compared to columns 1/7, mock-inf., and 2/8 DMSO control of infection). Importantly, the two conditions of pre-post- (columns 1–6) and post-incubation (columns 7–12) did not show basic differences. Thus, the HCMV-inhibitory efficacy of the compounds CCT and MBV were not enhanced through the pre-incubation of cells prior to infection. This result strongly suggests that the CDK8 inhibitor CCT does not act at the stage of virus entry.

Second, we were interested in the question of whether CDK8 might be involved in the regulation of viral nuclear capsid egress and the formation of the heterodimeric core nuclear egress complex (NEC) pUL50–pUL53. This aspect was stimulated by our earlier findings that a number of protein kinases can be associated with the HCMV-specific NEC [51,52,53,54,55,56]. As far as CDK8 is concerned, however, our previous analyses with NEC-specific cross-linking approaches suggested that CDK8, in contrast to CDK1, CDK2, and the vCDK/pUL97, might not belong to the group of NEC-associated protein kinases [52]. To this end, we performed an analysis of core NEC formation in HCMV-infected HFFs, based on IF staining and confocal imaging, in the presence or absence of partially effective concentrations of CDK8 inhibitors. In this assay, each of the CDK8 inhibitors, CCT, MSC, BI, or the vCDK/pUL97 inhibitor MBV as a NEC-targeting positive control, was incubated on the HCMV-infected cells (using HCMV AD169-UL50-HA for HA-tag-specific detection of the major NEC protein pUL50; Figure 5B). In the solvent-treated control (DMSO), the typical nuclear rim formation of the viral pUL50–pUL53 core NEC could be detected (Figure 5B, images 5–8). Upon treatment with MBV, a clear disruption of the smooth rim was observed, towards a speckled appearance of mislocalized NEC signals, as described elsewhere [52,56,57]. Upon treatment with either of the tree CDK8 inhibitors, no sign of a similarly NEC relocalization or disruption was detected. In these cells, the viral core NEC remained unaltered under the conditions of partial CDK8 inhibition. This finding supports our earlier statement that CDK8 does not contribute to the HCMV-specific NEC regulation.

Third, an analysis of virus progeny production and release was performed. Our expectation was that the measured effect of CDK8 inhibitors on the reduction in viral reporter signals, protein production, and viral spread in cultured cells, should likewise translate into a reduction in the release of infectious progeny. To address this question, media supernatant samples were collected from HCMV-infected HFFs, over a period of five viral replication cycles, from 2 d to 14 d p.i. (Figure 5C). Note that, also in this approach, partially effective drug concentrations of 5 nM, 2 nM, or 0.7 nM were applied in order to allow for an intermediate-level efficiency of virus production and release, in order to follow-up the residual viral replication kinetics. In all samples, i.e., treatment with CCT, MSC, BI, or the DMSO control, an initial onset of virus production was indicated by 4 d p.i. as expected (i.e., following the first 3 d round of intracellular viral replication). Notably, all three CDK8 inhibitors effected a reduction in the release of HCMV genome equivalents, thus indicating that virus production was constantly inhibited to the intended degree, along the replication kinetic up to 14 d p.i. (Figure 5C, right part of the curves 7–14 d p.i.). Statistically significant differences between DMSO and CDK8 inhibitors were found at 4, 7, 9, 11, and 14 d p.i.. This finding provides evidence that CDK8 inhibitors exert a rate-limiting effect onto virus production and release.

### 3.5. Assessment of Antiviral Drug Combination Treatment Using CDK8 Inhibitors and vCDK/pUL97 Inhibitor MBV in HCMV-Infected Cells

**Figure 6:**
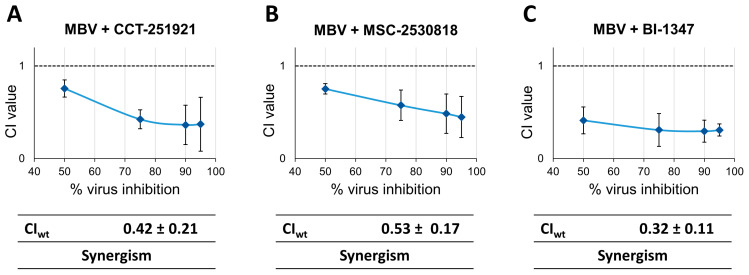
Antiviral drug combination treatment using Loewe additivity fixed-dose assay. The analysis included the (**A**) combination of MBV + CCT-251921, (**B**) combination of MBV + MSC-2530818, and (**C**) combination of MBV + BI-1347. A modified protocol of the HCMV-GFP replication system was used, and cells were treated with either single compounds, compound combinations (MBV + CDK8 inhibitor CCT, MSC, or BI, at the concentration ratio of 1:1000), or a solvent control. Antiviral efficacy (mean of quadruplicate measurements of biological duplicates) was expressed as a percentage of the solvent control and analyzed using the CompuSyn software. Only experiments with an r value > 0.90 and EC50 values near previously determined concentrations were accepted.

Due to the fact that both CDK8 and vCDK/pUL97 have regulatory importance for the HCMV replication efficiency in cultured cell models, we addressed the question of their putative synergistic potential upon combinatorial drug cotreatment. In this approach, we applied the Loewe additivity fixed-dose assay, as previously established [14]. The cotreatment of HCMV-infected HFFs was performed with combinations MBV + CCT, MBV + MSC, and MBV + BI (Figure 6). The range of effective concentrations of the two drugs was determined by single-drug treatment experiments before. In all three combinations, the results indicate a clear-cut drug synergism, with weighted combination indices (CI_wt_) of 0.42 ± 0.21, 0.53 ± 0.17, and 0.32 ± 0.11 (Figure 6, panels left to right). Thus, the drug combination strategy directed to CDK8 and vCDK/pUL97 appears highly promising. Of note, this option has already been suggested by earlier reports that demonstrated synergistic antiviral combinations between various CDK and vCDK/pUL97 inhibitors [12,58]. In the present study, we confirmed this phenomenon of CDK + vCDK inhibitor synergism for the specific example of CDK8 inhibitors. The strong synergistic potential of the MBV combinations with CCT, MBV, or BI was further illustrated by a second experimental setting (applying very low-passage HFFs, using a 12-well instead of the 96-well format). Also, this experimental setting yielded very distinct CI_wt_ values that consistently pointed to drug synergism (Figure S7). Thus, the very stringent, nanomolar-range anti-HCMV efficacy of selective CDK8 inhibitors, together with their potential to allow for synergistic antiviral drug interactions, indicates the to date unaddressed therapeutic options of antiviral treatment and combinatorial drug cotreatments.

### 3.6. Broad-Spectrum Antiviral Activity of CDK8 Inhibitors against Human and Animal α-/β-/γ-Herpesviruses as Well as Non-Herpesviruses

In regard of the question of whether CDK8 constitutes a host factor with importance for many viruses, and thus whether CDK8 inhibitors may exert some broader antiviral activity, we performed a comparative analysis. Our expectation to see a wider-ranging type of activity was based, on the one hand, by the recently raised understanding that specifically the CDK8/mediator complex plays a virus-supportive role for HCMVas well as related and nonrelated human viruses [22]. On the other hand, earlier findings provided numerous examples, which illustrated that CDKs are generally integrated into viral replication cycles, and CDK–cyclin complexes themselves were partly reprogrammed through the infection for the benefit of viral productivity [59,60,61,62,63]. Against this background of knowledge, we underwent a broader antiviral study of CDK8 inhibitor sensitivity. The approach included a panel of 11 selected α-, β-, and γ-herpesviruses (2, 7, and 2, respectively) as well as 2 non-herpesviruses (Table 3; Table 4; Figure S4). This antiviral drug assessment showed that, among the 11 selected herpesviruses used, the 6 human and animal CMVs had an excellent drug sensitivity, with EC_50_ values in the nanomolar or submicromolar range (i.e., HCMV, RhCMV, ChCMV, MCMV, RCMV, and GPCMV; Table 3; Figures S1 and S2). Interestingly, another human β-herpesvirus, namely HHV-6A, showed a comparably modest sensitivity at micromolar concentrations (CCT) or no sensitivity up to 50 µM (MSC, BI; Table 4, right; Figure S3). Likewise, the α- and γ-herpesviruses analyzed showed either no sensitivity in the concentration range of interest, or for some of the three CDK8 inhibitors, a limited sensitivity in the micromolar/submicromolar range (HSV-1, VZV, and KSHV; Table 4; Figure S3; in addition, EBV also showed low sensitivity). Interestingly, the murine γ-herpesvirus MHV-68 showed a markedly stronger drug sensitivity at nanomolar concentrations, as analyzed in Vero cells, for reasons that to date remain unexplained (Table 4; Figure S3). Moreover, drug sensitivity was also determined for the two non-herpesviruses analyzed, where HAdV showed comparably lower EC_50_ values than JCPyV (Table 4, right panel; Figure S4). This finding clearly illustrates the very pronounced dependency of human and animal CMVs on CDK8 activity, and the broad-spectrum antiviral efficacy of CDK8 inhibitors, either manifested in human or animal host cells. This finding from the comparative analysis underlines that CDK8 plays a conserved virus-supportive role, especially in the replication of CMVs, and moreover, that CDK8 inhibitors are able to target non-human homologs of CDK8. On the other hand, some limited or even lack of sensitivity of specific α-, β-, and γ-herpesviruses and non-herpesviruses may indicate specific differences in the interaction of CDK8 with regulatory processes and/or viral proteins. The understanding of these mechanistic aspects should be deepened in subsequent studies. Combined, the new findings support our concept of a profound relevance of CDK8 for the replication of CMVs, and, in some parts, additional viruses in cultured cell models. This situation may foster novel opportunities for both the CMV-specific antiviral potential as well as the crossviral inhibitory potential of CDK8 inhibitors.

### 3.7. Conclusions: Specific Focus on a Future Use of CDK8 Inhibitor as a Potential Antiviral Drug

Recently, the work of our group and other investigators pointed to a promising option of utilizing CDK inhibitors as potential antiviral drugs or constituents of drug combination regimens [12,13,61,62,64,65]. As far as CDK8 is concerned, as a postulated target of new antiherpesviral therapy, our findings related to the investigated CDK8 compound (SEL120), developmental drugs (compounds of the present study, CCT, MSC, and BI), as well as approved CDK inhibitors (abemaciclib) unequivocally demonstrate the inherent power of this approach. Concerning the question of the clinical application of CDK8 inhibitors, it should be emphasized that novel chemical formulations and drugs are currently being developed [66,67,68]. The studies of clinical use of CDK8 inhibitors span the field of anti-cancer approaches, such as acute myeloid leukemia [69], prostate cancer metastasis [70], and other solid tumors [71], as well as a drug-mediated antagonization of human immunodeficiency virus 1 (HIV-1) reactivation [72]. According to the presently available clinical data, the drug safety of CDK8 inhibitors is generally promising, and treatment-related adverse events (AEs) are few, e.g., limited to diarrhea or weakness. Importantly, the data published to date, which describe a significant inhibition of tumor cell progression and migration, provide a promising view towards successful drug development [70,71].

Two main questions may then arise from the CDK8-based antiviral concept, namely (i) how to consider the chances of pharmacological development of an antiviral CDK inhibitor and (ii) how to define the mechanistic role that CDK8 plays in its virus-supportive function of viral replication. As far as drug development is concerned, it is very striking to see the vast achievements CDK inhibitors have made in clinical use in general, i.e., first of all in anticancer applications [49,73]. The transfer of the clinical use of CDK inhibitors to other areas, such as a broader use in therapy of inflammation disease, virus infection, or metabolic defects, is still a matter that needs to be clarified in terms of individual drug candidates. With the approval of the vCDK inhibitor MBV against HCMV disease, a first hallmark was set up that may consequently stimulate further drug developments. In regard of the antiviral MoA of CDK8 inhibitors, several details still have to be deciphered. To date, we have clear experimental indications that, similar to CDK7 inhibitors [13,17,28], also in the case of CDK8 inhibitors, a multifaceted impact on virus replication is exerted, specifically in the case of HCMV. It is obvious that the relevance of CDK8 for transcriptional regulation is an integral part of viral transcription in infected cells [22]. Beyond that, we have initial evidence for a specific regulatory impact by the direct CDK8-mediated phosphorylation of viral proteins (Marschall, Obergfäll et al., unpublished results), similar to the experimental details already provided for CDK7 [28]. The current stage of experimental evidence points to the phosphorylation of a subset of cytomegaloviral early regulators by CDK8, and moreover, it can be speculated that our additional evidence of the strict nuclear localization of host CDK8 in HCMV-infected primary fibroblasts may point to an influence on the regulated nuclear phase of herpesviral replication. Such an accessory regulatory function of CDK8 may concern a support in viral nuclear transport and replicative processes, which will be the subject of future analyses.

Finally, which innovative benefits may be provided by a host-directed antiviral strategy (HDA) based on CDK8 inhibitors remains an open question. Here, a main point of importance could be explained by the increase in the viral drug resistance barrier, as compared to conventional direct-acting antivirals (DAAs). In the case of HCMV, drug resistance is a limiting factor of all approved DAAs to date [74]. In the fields of antiretroviral and antihepatitisviral therapies, however, the implementation of HDAs has recently provided solid progress in terms of a reduction in drug resistance issues [75,76,77,78]. Thus, a clear stipulation, towards specific pharmacological improvements utilizing the CDK8-based single-drug or combined-drug treatment options, can be considered by the present state of data. In particular, the latter point of combined-drug synergies may be addressed in greater detail. We described the statistically significant anti-HCMV efficacy using HDA + DAA combination treatment in vitro, i.e., the very promising combinatorial effect between CDK8 inhibitor and MBV. In a very similar manner, we recently reported on drug synergy between various CDK/vCDK inhibitor combinations, e.g., using CDK7 + vCDK/pUL97-directed drugs, comprising synergistic levels of anti-HCMV activity [12,13]. These options of combination treatment might also open up opportunities unexploited to date with mechanistically different antiherpesviral drugs. This point might involve CDK and vCDK inhibitors, on the one hand, as well as nucleoside analogs, terminase inhibitors, helicase inhibitors, and others, on the other hand. All in all, the multifaceted chances of drug targeting, the various MoAs, and the combinatorial treatment options should argue for a further advancement of the CDK8-based antiviral concept.

## Figures and Tables

**Table 1:** Anti-HCMV activity of the selected CDK inhibitors (µM).

Target	Compound	EC_50_ ^a^	CC_50_^b^	SI ^c^
vCDK/pUL97	MBV	0.35 ± 0.42	>100	>250
CDK2	CDK2 Inh II	6.33 ± 2.80	>100	>16
CDK7	LDC4297	0.009 ± 0.002	>10	>1000
CDK8	SEL120	0.079 ± 0.001	5.90 ± 3.85	75

^a^ Half-maximal efficacy (EC_50_) against HCMV AD169-GFP was determined via the GFP-based replication assay; these EC_50_ values represent the mean ± SD of ≥2 biological replicates. ^b^ Half-maximal cytotoxicity in mock-infected HFFs was determined via the Neutral Red assay; these CC_50_ values represent the mean ± SD of ≥biological replicates. ^c^ Selectivity index was calculated as the ratio CC_50_/EC_50_. EC_50_ and EC_50_ values are presented in micromolar concentrations.

**Table 2:** Determination of antiviral EC_50_ values of CDK8 inhibitors for three HCMV strains in two different cell types *.

		EC_50_ (nM)			CC_50_ (µM)
	Compound	AD169-GFP^1^	TB40-YFP^2^	Merlin-GFP^3^	Mock-inf.
HFF	CCT-151921	3.90 ± 2.00	10.00 ± 6.33	1.27 ± 1.17	>100
	MSC-2530818	1.21 ± 0.77	4.97 ± 3.30	5.55 ± 5.63	>100
	BI-1347	0.93 ± 0.67	2.83 ± 2.30	0.54 ± 0.99	>100
U373	CCT-151921	16.33 ± 7.13	8.50 ± 11.13	14.67 ± 16.33	>100
	MSC-2530818	18.47 ± 11.20	12.33 ± 11.33	4.47 ± 4.40	87 ± 6
	BI-1347	10.80 ± 5.73	19.33 ± 21.00	1.40 ± 1.37	>100

* The antiviral efficacy of CDK8 inhibitors was assessed for HCMV AD169-GFP^1^, TB40-YFP^2^, and Merlin-GFP^3^-based replication assay in HFFs and U373 cells by the measurement of samples in technical quadruplicates derived from ≥2 biological replicates. Note that the different ranges of EC_50_ and CC_50_ values are presented in nanomolar (nM) or micromolar concentrations (µM), respectively.

**Table 3:** Antiviral EC_50_ values of CDK8 inhibitors against selected human and animal β-herpesviruses.

	Antiviral Drug Efficacy Comparing Human and Animal Cytomegaloviruses *					
Compound	HCMV (nM)	RhCMV (nM)	ChCMV (nM)	MCMV (nM)	RCMV (nM)	GPCMV (nM)
CCT-151921	3.90 ± 2.00	2.48 ± 1.63	31.00 ± 18.00	2.65 ± 4.50	11.17 ± 22.67	19.42 ± 33.18
MSC-2530818	1.21 ± 0.77	2.17 ± 1.37	21.00 ± 26.50	1.80 ± 1.99	4.87 ± 4.67	4.48 ± 4.84
BI-1347	0.93 ± 0.67	0.40 ± 0.48	11.55 ± 22.65	1.57 ± 1.71	1.08 ± 1.49	1.11 ± 2.37

* Mean values ± SD of quadruplicate measurements, as derived from determinations in the numbers of biological replicates indicated; viruses and cells were used as follows: human cytomegalovirus (HCMV AD169-GFP) in HFFs [nM], *n* = 3; rhesus cytomegalovirus (RhCMV-GFP) in HFFs [nM], *n* = 2; chimpanzee cytomegalovirus (ChCMV strain Herberling) in HFFs [nM], *n* = 2; murine cytomegalovirus (MCMV-GFP) in MEFs [nM], *n* = 2; rat cytomegalovirus (RCMV-mCherry) in REFs [nM] *n* = 3; guinea pig cytomegalovirus (GPCMV-GFP) in GPEFs [nM], *n* = 1. In the case of GPCMV-GFP infection, antiviral drug activity was determined in two ways, by GFP quantitation using the Victor X4 microplate reader and by Alamar Blue cell viability assay for measuring virus-induced cytopathogenicity, in order to obtain a reproduction of the data. For primary data of all viruses analyzed, see detailed panels in Figures S1–S4. Note that the range of individual EC_50_ values is provided in nanomolar concentrations.

**Table 4:** Antiviral EC_50_ values of CDK8 inhibitors against α-, β-, and γ-herpesviruses and non-herpesviruses.

	Antiviral Drug Efficacy Comparing a Panel of Selected Herpes- and Non-Herpesviruses *						
	α-Herpesviruses		β-Herpesviruses	γ-Herpesviruses		Non-Herpesviruses	
Compound	HSV-1 (µM)	VZV (µM)	HHV-6A (µM)	KSHV (µM)	MHV-68 (µM)	HAdV (µM)	JCPyV (µM)
CCT-151921	>50	28.00 ± 9.00	39.00 ± 21.00	0.30 ± 0.95	0.022 ± 0.019	0.03 ± 0.03	3.67 ± 3.00
MSC-2530818	42.30 ± 4.90	>50	>50.00	>40	0.0025 ± 0.003	0.34 ± 0.26	0.14 ± 3.53
BI-1347	>50	26.00 ± 10.00	>50.00	0.31 ± 0.61	0.0008 ± 0.001	0.05 ± 0.11	0.86 ± 1.55

* Mean values ± SD of quadruplicate measurements, as derived from determinations in the numbers of biological replicates indicated; viruses and cells were used as follows: herpes simplex virus type 1 (HSV-1 166v VP22-GFP) in HFFs [µM], *n =* 2; VZV, varicella zoster virus (VZV Oka-GFP) in HFFs [µM], *n =* 2; human β-herpesvirus 6 (HHV-6A-GFP) in J-Jhan cells [µM], *n =* 3; Kaposi’s sarcoma-associated herpesvirus (KSHV rKSHV.219-GFP) in iSLK.219 cells [µM], *n =* 2; murine γ-herpesvirus 68 (MHV-68-Luc) in Vero cells [nM], *n =* 2; human adenovirus (HAdV species C5 DBP-mneongreen) in A549 cells [µM], *n =* 2; human polyomavirus type 2 (JCPyV strain Mad-4) in COS-7 cells [µM], *n =* 1. Note that the range of individual EC_50_ values is provided in micromolar concentrations.

## Data Availability

The original contributions presented in the study are included in the article/Supplementary Material, further inquiries can be directed to the corresponding author.

## Supplementary Materials

The following supporting information can be downloaded at: https://www.mdpi.com/article/10.3390/pharmaceutics16091238/s1. Figure S1. Anti-HCMV activity of the three CDK8-inhibitory developmental small molecules CCT-151921, MSC-2530818, and BI-1347. (Additional primary data panels, in the context of Figure 1; Figure 2, as indicated in the text description). Figure S2. Antiviral activity of the CDK8 inhibitors CCT-151921, MSC-2530818, and BI-1347, determined against a selection of animal CMVs. (Additional primary data panels, in the context of Table 3, as indicated in the text description). Figure S3. Antiviral activity of the CDK8 inhibitors CCT-151921, MSC-2530818, and BI-1347, determined against various herpesviruses. (Additional primary data panels, in the context of Table 3; Table 4, as indicated in the text description). Figure S4. Antiviral activity of the CDK8 inhibitors CCT-151921, MSC-2530818, and BI-1347, determined against human non-herpesviruses. (Additional primary data panels, in the context of Table 4, as indicated in the text description). Figure S5. Analysis of transfection-mediated intracellular CDK8-specific siRNA knock-down (KD) in primary fibroblasts. (Additional primary data panels, in the context of Figure 4, as indicated in the text description.) Figure S6. Analysis of transfection-mediated intracellular CDK8-specific siRNA knock-down (KD) in U373 cells. (Modified experimental setting, in the context of Figure 4, as indicated in the text description). Figure S7. Antiviral drug combination treatment using Loewe additivity fixed-dose assay. (Modified experimental setting, in the context of Figure 6, as indicated in the text description).
