# Supplementary material for: Cyclin-Dependent Kinase 8 Represents a Positive Regulator of Cytomegalovirus Replication and a Novel Host Target for Antiviral Strategies
Source: Pharmaceutics. 2024 Sep 23;16(9):1238. doi: 10.3390/pharmaceutics16091238 (PMC11435438; doi:10.3390/pharmaceutics16091238)
Supplement: Supplementary file 1 [file pharmaceutics-16-01238-s001.zip › pharmaceutics-3190225-supplementary.pdf]

pharmaceutics

Supplementary Materials

Cyclin-Dependent Kinase 8 Represents a Positive Regulator of Cytomegalovirus Replication and a Novel Host Target for Antiviral Strategies

Debora Obergfäll [1,$], Markus Wild [1,$], Mona Sommerer [1], Malena Barillas Dahm [1], Jintawee Kicuntod [1], Julia Tillmanns [1], Melanie Kögler [1], Josephine Lösing [1], Kishore Dhotre [1], Regina Müller [1], Christina Wangen [1], Sabrina Wagner [1], Quang V. Phan [2,3], Lüder Wiebusch [2], Katarína Briestenská [4,5], Jela Mistríková [4,5], Lauren Kerr-Jones [6], Richard Stanton [6], Sebastian Voigt [7], Friedrich Hahn [1], Manfred Marschall [1,*]

[1] Institute for Clinical and Molecular Virology, Friedrich-Alexander University of Erlangen-Nürnberg (FAU), Schlossgarten 4, 91054 Erlangen, Germany; debora.obergfaell@fau.de, markus-wild@mail.de, mona.sommerer@googlemail.com, jintawee.kicuntod@extern.uk-erlangen.de, jul.tillmanns@fau.de, melanie.koegler@uk-erlangen.de, josi.loesing@fau.de, kishore.dhotre@uk-erlangen.de, malena.barillas@fau.de, mueller.regina@uk-erlangen.de, christina.wangen@uk-erlangen.de, sabrina.wagner@uk-erlangen.de, friedrich.hahn@uk-erlangen.de, manfred.marschall@fau.de
[2] Charité – University Medicine Berlin, Clinics for Pediatry, Onkology and Hematology, Campus Virchow-Klinikum, Augustenburger Platz 1, Forum 4, 13351 Berlin, Germany; lueder.wiebusch@charite.de
[3] Richard Sherwood Laboratory, Brigham and Women's Hospital, Harvard Medical School, 77 Avenue Louis Pasteur, Boston, MA 02115, USA; qvphan@bwh.harvard.edu
[4] Department of Microbiology and Virology, Faculty of Natural Sciences, Comenius University in Bratislava, Ilkovičova 6, 842 15 Bratislava, Slovak Republic; katarina.briestenska@savba.sk, jela.mistrikova@savba.sk
[5] Institute of Virology, Biomedical Research Center, Slovak Academy of Sciences, Dúbravská cesta 9, 845 05 Bratislava, Slovak Republic; katarina.briestenska@savba.sk, jela.mistrikova@savba.sk
[6] Division of Infection & Immunity, School of Medicine, Cardiff University, Henry Wellcome Building, Heath Park, Cardiff CF14 4XN, UK; StantonRJ@cardiff.ac.uk, KerrLE@cardiff.ac.uk
[7] University Clinical Center Essen (Universitätsklinikum, AöR), Institute for Virology, Virchowstr. 179, 45147 Essen, Germany; sebastian.voigt@uk-essen.de
* Correspondence: e-mail manfred.marschall@fau.de; phone +49 9131 85-36096
$ These authors contributed equally to the study

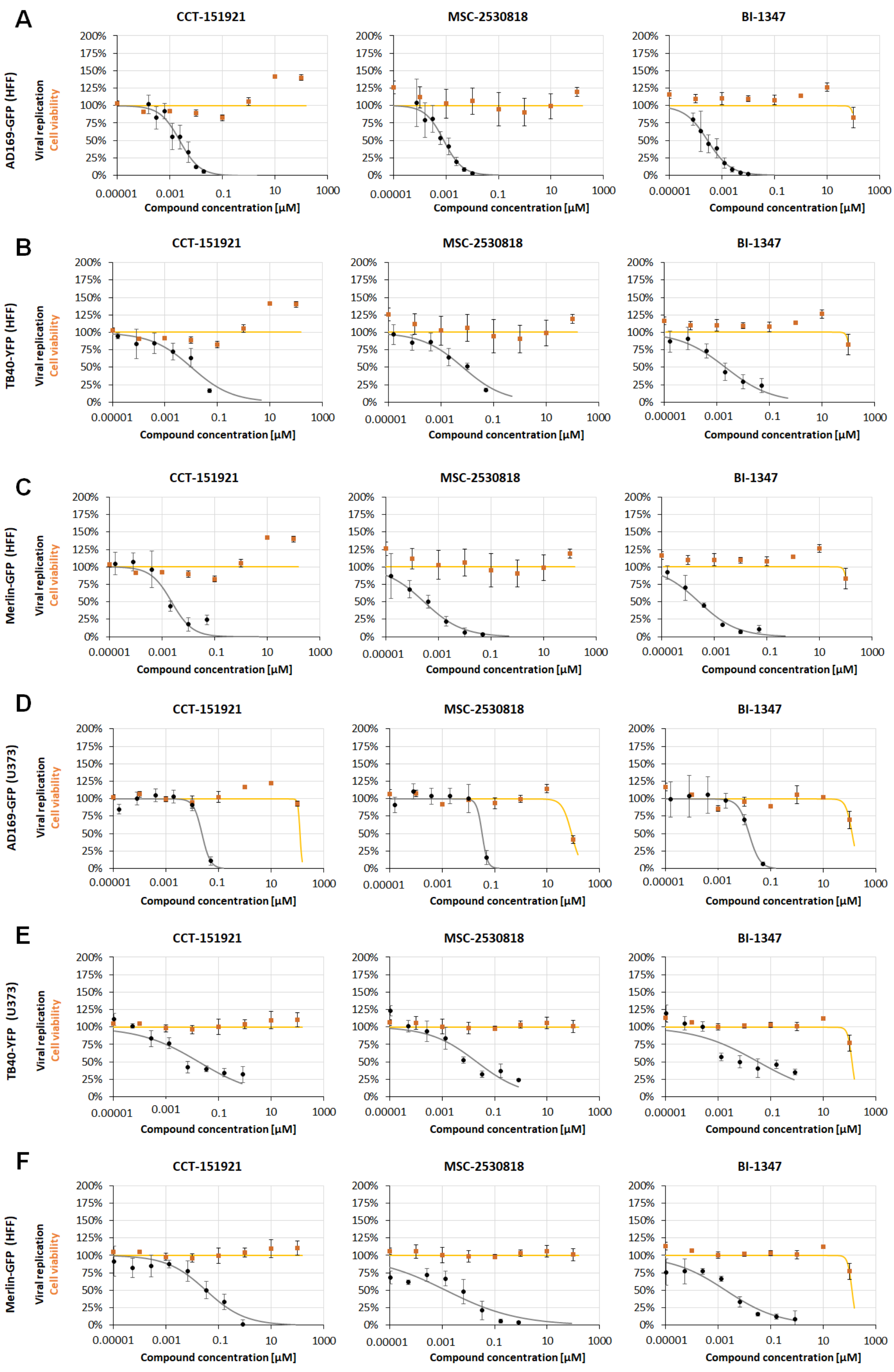

Figure S1. Anti-HCMV activity of the three CDK8-inhibitory developmental small molecules CCT-151921, MSC-2530818, and BI-1347. Antiviral activity was quantitatively assessed against **(A)** AD169-GFP, **(B)** TB40-YFP, and **(C)** Merlin-GFP, in HCMV-infected HFFs, and against **(D)** AD169-GFP, **(E)** TB40-YFP, and **(F)** Merlin-GFP, in HCMV-infected U373 cells. The compounds were administered immediately post-infection (p.i.), beginning at a concentration of 50 nM, then followed by serial dilution steps, in order to attain the range of effective concentrations as finally indicated. Cells were fixed at 7 d p.i. for quantitative GFP fluorometry. Additionally, cell viability was assessed for uninfected cells using the NRA. Values represent mean ± SD of measurements in triplicate (NRA) or quadruplicate (GFP), with panels depicting one representative experiment out of ≥ three independent replicates.

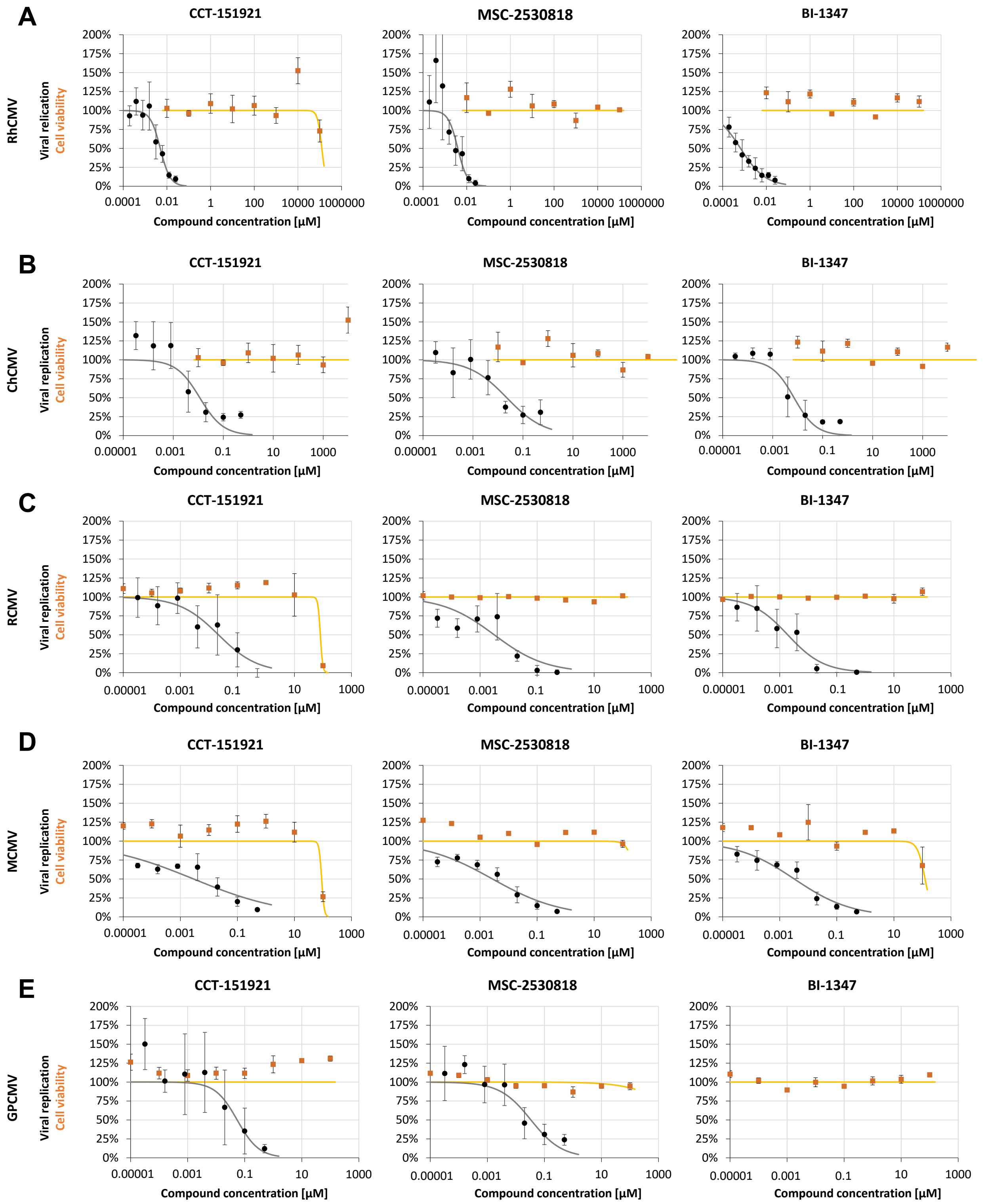

Figure S2. Antiviral activity of the CDK8 inhibitors CCT-151921, MSC-2530818, and BI-1347, determined against a selection of animal CMVs. Antiviral activity was quantitatively assessed against **(A)** rhesus cytomegalovirus (RhCMV-GFP) in HFFs, **(B)** chimpanzee cytomegalovirus (ChCMV strain Herberling) in HFFs, **(C)** rat cytomegalovirus (RCMV-mCherry) in REFs, **(D)** murine cytomegalovirus (MCMV-GFP) in MEFs, **(E)** guinea pig cytomegalovirus (GPCMV-GFP) in GPEFs. The compounds were administered immediately p.i.. Cells were fixed for quantitative GFP or RFP fluorometry. Additionally, cell viability was assessed for uninfected cells using the NRA. Values represent mean ± SD of measurements in triplicate (NRA) or quadruplicate (GFP), with panels depicting one representative experiment out of ≥ three independent replicates. For ChCMV, cells were immunostained with Anti-Cytomegalovirus Antibody Alexa Fluor™ 488, and IF signals were measured using the ImageXpress Pico device.

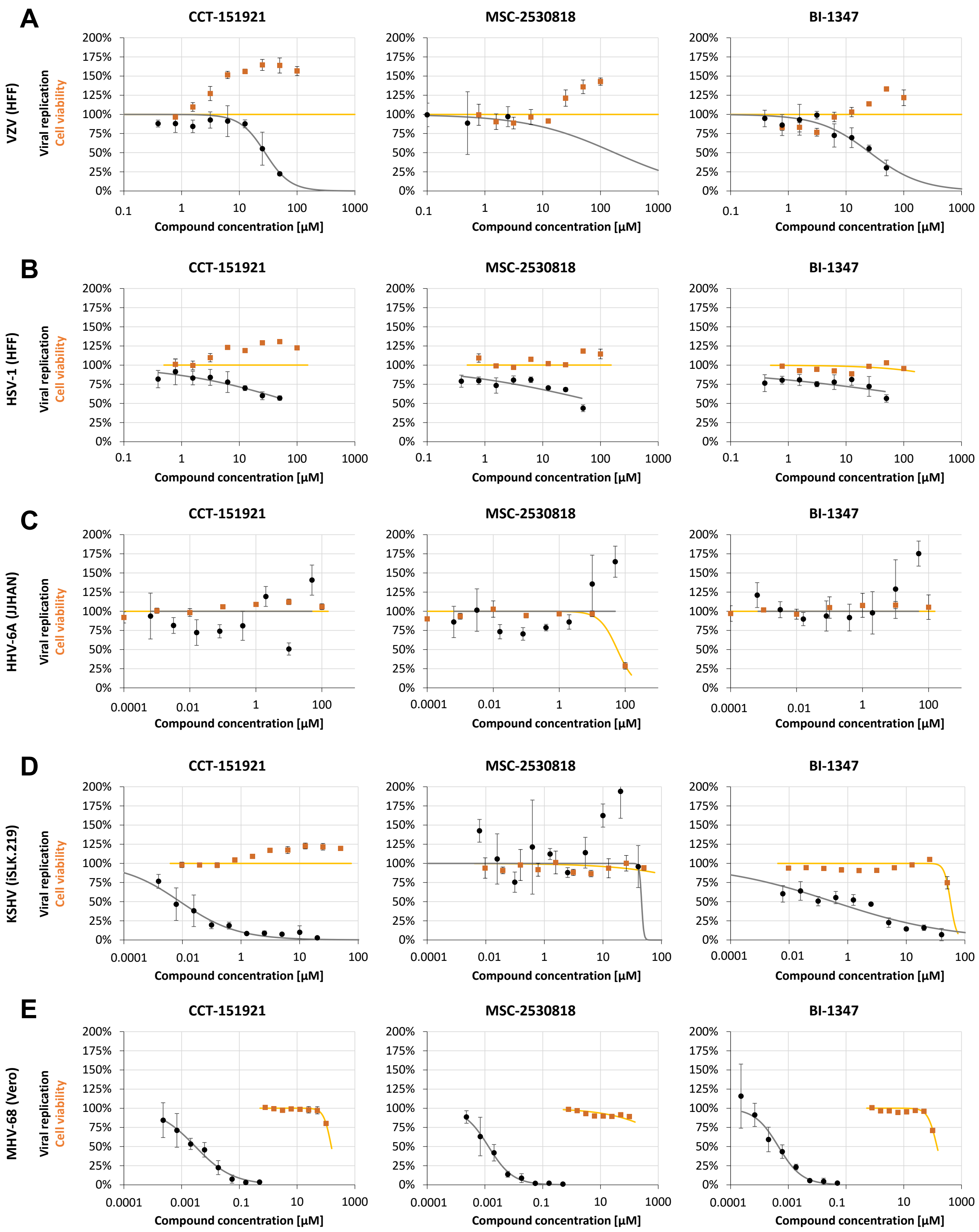

Figure S3. Antiviral activity of the CDK8 inhibitors CCT-151921, MSC-2530818, and BI-1347, determined against various herpesviruses. Antiviral activity was quantitatively assessed against **(A)** varicella zoster virus (VZV Oka-GFP) in HFFs, **(B)** herpes simplex virus type 1 (HSV-1 166v VP22-GFP) in HFFs, **(C)** human herpesvirus 6A (HHV-6A-GFP) in J-Jhan cells, **(D)** Kaposi's sarcoma-associated herpesvirus (KSHV rKSHV.219-GFP) in iSLK.219 cells, and **(E)** murine g-herpesvirus 68 (MHV-68-Luc) in Vero cells. The compounds were administered immediately p.i., and cells were fixed before quantitative GFP fluorometry was performed. Additionally, cell viability was assessed for uninfected cells using the NRA. Values represent mean ± SD of measurements in triplicate (NRA) or quadruplicate (GFP), with panels depicting one representative experiment out of ≥ three independent replicates.

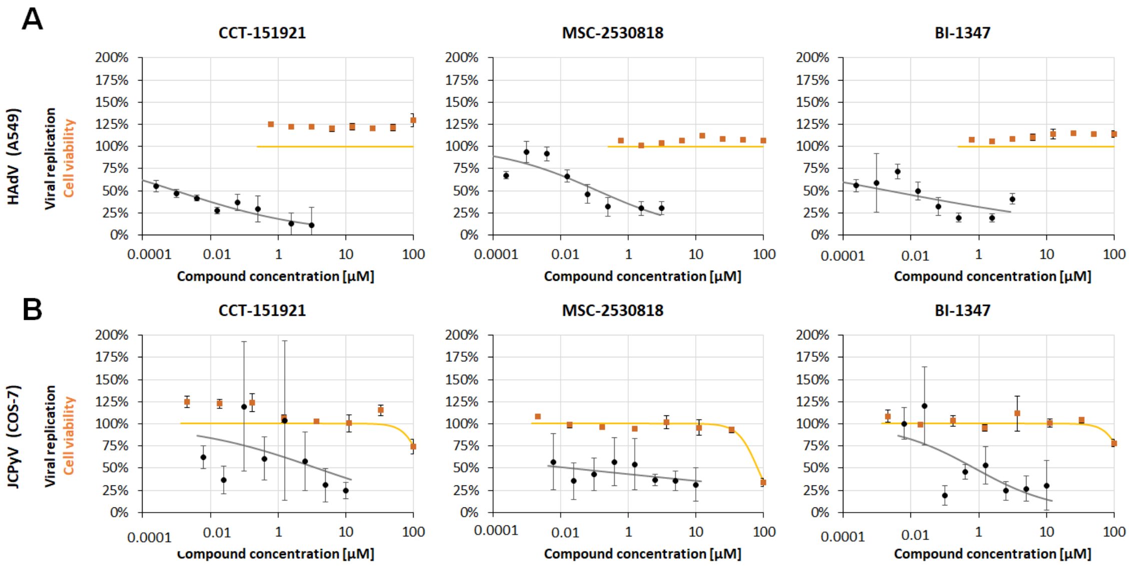

Figure S4. Antiviral activity of the CDK8 inhibitors CCT-151921, MSC-2530818, and BI-1347, determined against human non-herpesviruses. Antiviral activity was quantitatively assessed against **(A)** human adenovirus (HAdV species C5 DBP-mneongreen) in A549 cells, and **(B)** human polyomavirus type 2 (JCPyV strain Mad-4) in COS-7 cells. The compounds were administered immediately p.i., and cells were fixed before quantitative GFP fluorometry was performed (HAdV), or total DNA was isolated before virus-specific qPCR was performed (JCPyV). Additionally, cell viability was assessed for uninfected cells using the NRA. Values represent mean ± SD of measurements in triplicate (NRA) or quadruplicate (GFP), with panels depicting one representative experiment out of ≥ three independent replicates.

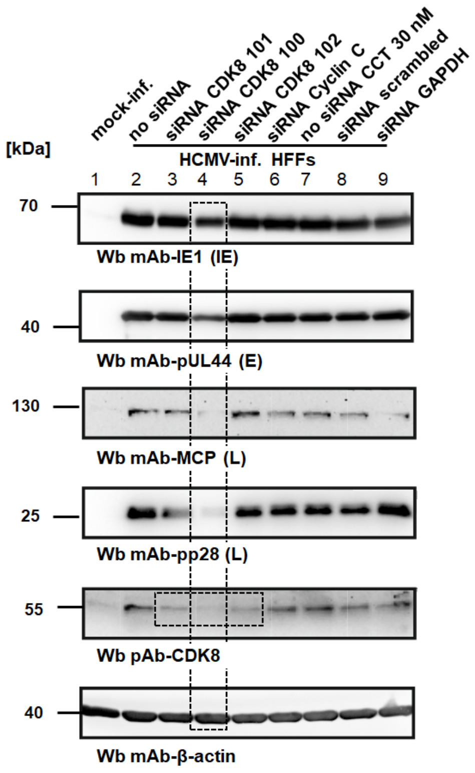

Figure S5: Analysis of transfection-mediated intracellular CDK8-specific siRNA knock-down (KD) in primary fibroblasts. (A) (**A**) An experimental siRNA KD was performed using the three targeted siRNAs CDK8 100, CDK8 101, CDK8 102, plus siRNA Cyclin C (as a non-CDK8-targeted control) for tranfection (Lipofectamine™ 3000), at a final concentration of 10 pmol/well. HFFs were cultivated in 6-well plates, and were infected at one d post-siRNA transfection with HCMV AD169 at MOI of 1.0. As additional control samples, mock-infected cells, mock-transfected (no siRNA) cells, mock-transfected cells treated with CCT-151921, a non-specific siRNA (scrambled), and a GAPDH-specific siRNA were used. Cells were harvested at 7 d p.i. for the preparation of total lysates, to be analyzed by SDS-PAGE/Wb procedures. Wb antibody staining was performed **(A)** for viral IE1p72, pUL44, MCP and pp28 as representative viral immediate early (IE), early (E), and late proteins (L), respectively. In addition, cellular CDK8 (siRNA target) as well as β-actin (house-keeping protein and loading control) were stained in parallel.

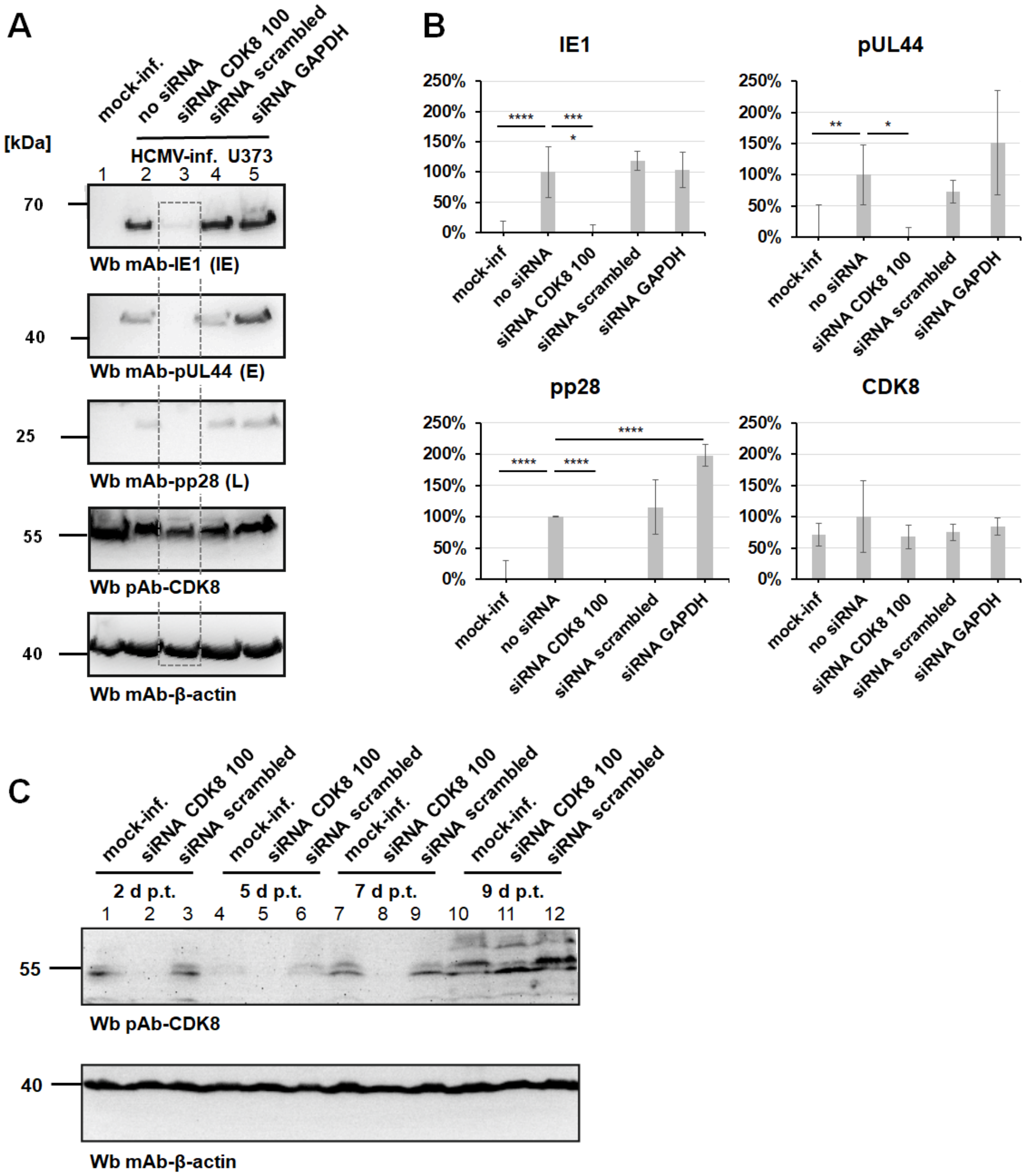

Figure S6. Analysis of transfection-mediated intracellular CDK8-specific siRNA knock-down (KD) in U373 cells. (A) The CDK8-specific siRNA KD was performed under optimized conditions (Lipofectamine™ RNAiMAX), using the siRNAs CDK8 100 at a final concentration of 10 pmol/well**.** U373 cells were cultivated in 6-well plates and were infected with HCMV AD169 at MOI 0.1 at 2 d post-siRNA transfection. As control samples, mock-infected cells, mock-transfected (no siRNA) cells, a non-specific siRNA (scrambled), and a GAPDH-specific siRNA were used. Cells were harvested at 7 d p.i. for the preparation of total lysates, to be analyzed by SDS-PAGE/Wb procedures**. (B)** Densitometric analysis was performed in quadruplicate measurements (SDS-PAGE/Wbs in duplicate, densitometry in duplicate) using AIDA image analyzer, and statistical analysis was done using Anova followed by Bonferoni for multiple comparison (* $P \leq 0.05$ ** $P \leq 0.01$ *** $P \leq 0.001$ **** $P \leq 0.0001$). **(C)** The CDK8-specific siRNA KD was performed (Lipofectamine™ RNAiMAX), using the siRNAs CDK8 100 at a final concentration of 10 pmol/well**.** U373 cells were cultivated and transfected in 6-well plates, before cell samples were harvested at 2, 5, 7, and 9 d post-siRNA transfection. As control samples, mock-infected cells and non-specific siRNA (scrambled) were used. In additiona, total cell samples harvested at 7 d p.i. were also used for the preparation of total lysates, to be analyzed by SDS-PAGE/Wb procedures, with stainings as indicated.

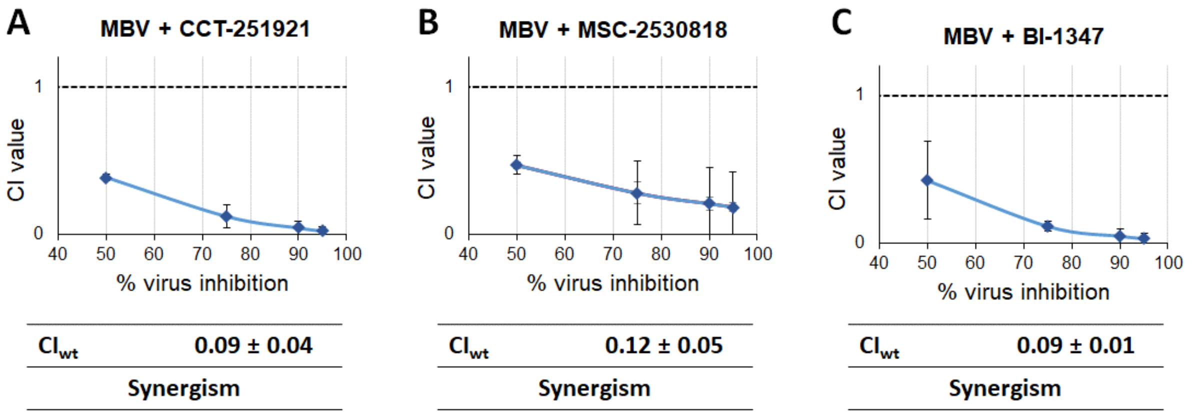

Figure S7. Antiviral drug combination treatment using Loewe additivity fixed-dose assay. The analysis included **(A)** combination of MBV + CCT-251921, **(B)** combination of MBV + MSC-2530818, and **(C)** combination of MBV + BI-1347. A modified protocol of the HCMV-GFP replication system was used and cells were treated with either single compounds, compound combinations (MBV + CDK8 inhibitor CCT, MSC, or BI, at the concentration ratio of 1:1000), or a solvent control. Antiviral efficacy (mean of quadruplicate measurements of biological duplicates) was expressed as a percentage of the solvent control and analyzed using CompuSyn software. Only experiments with an r value > 0.90 and EC50 values near previously determined concentrations were accepted.
